# Effect of oral functional training on immunological abilities of older people: a case control study

**DOI:** 10.1186/s12903-017-0461-7

**Published:** 2018-01-08

**Authors:** Mitsue Sato, Masahiro Sugimoto, Yuko Yamamoto, Juri Saruta, Keiichi Tsukinoki

**Affiliations:** 1 Nursing Science, Ryotokuji University, Urayasu, Chiba, 279-8567 Japan; 2 Nursing Science, Human Care Department, Tohto College of Health Sciences, Fukaya, Saitama, 366-0052 Japan; 3 Department of Oral Science, Graduate School of Dentistry, Kanagawa Dental University, Yokosuka, Kanagawa 238-8580 Japan; 4 Institute for Advanced Biosciences, Keio University, Tsuruoka, Yamagata, 997-0052 Japan; 5 Health Promotion and Preemptive Medicine, Research and Development Center for Minimally Invasive Therapies, Tokyo Medical University, Shinjuku, Tokyo, 160-0022 Japan; 6 Department of Oral Science, Division of Salivary Gland and Health Medicine, Graduate School of Dentistry, Kanagawa Dental University, Yokosuka, Kanagawa 238-8580 Japan

**Keywords:** Immunoglobulin a, “Aged, 80 and over”, Saliva, Salivary gland, Exercise therapy

## Abstract

**Background:**

Oral functional ability decreases with age, and systemic immunological ability and quality of life can also deteriorate. Continuous moderate whole-body exercise for older people is known to improve oral functional and their immunological abilities. Here, we evaluated the effect of oral exercise as an alternative training method for highly older people who cannot perform whole-body exercises.

**Methods:**

Unstimulated whole saliva samples had been collected for three times before training as baseline data and one time after 3 and 6 weeks of training each. Participants were instructed to conduct self-massage; their tongues were used to press their orbicularis oris muscle and buccinators, and instructed to perform bilateral massage of three major glands for facilitating saliva secretion. Medical histories, daily life habits and characteristics were also collected.

**Results:**

Totally 30 participants (84.2 ± 8.5 years) were enrolled. In contrast to previous researches, increase in salivary Immunoglobulin A (IgA) after the training was not observed. Interestingly, hierarchical clustering analyses revealed clear individual variations as two prominent clusters and a strong positive correlation between stimulated saliva flow rate and IgA flow rate, regardless of the continuous oral functional exercise. Only body mass index (BMI) showed significant differences between the two groups (*Z* = 2.06, *P* = 0.039, Wilcoxon rank-sum test) among all collected parameters.

**Conclusion:**

Oral functional training limitedly effects on salivary parameters of highly older people. On the other hand, BMI characterized salivary features more than any other parameters, such as the presence of diseases or medication use in these people. Trial registration: UMIN-CTR Clinical Trial UMIN000028394 on 27/July 2017, retrospectively registered.

## Background

Older people are at high risk of infectious diseases due to reduction of immune system function, and the average age of onset of reduced immune system function has reportedly begun to decrease [1–3]. Improving immune system function in aging people is expected to contribute to improving their overall health status. Therefore, the effects of various systemic exercises on immune system in elder people have been investigated [4–7].

Oral cavity plays an important role in various functions, including respiration and other essential biological functions, including eating and swallowing, along with articulation, which contribute to quality of life, systemic health and communication [8]. It is well known the association between worse oral health in elderly subjects and higher respiratory infection, such as pneumonia and influenza virus infection, including upper–respiratory tract infections (URTI) [9, 10]. The associations between oral inflammatory disorders, such as gingivitis and periodontitis, and frailty and several diseases, atherosclerosis cardiovascular diseases, arthritis, and diabetes in older people [11]. Weakened oral cavity function, such as tooth loss, and lower oral hygiene in elder subjects reduces immune system function, and leads to decreased systemic health [12, 13]. Thus, many attempts of continuous oral care for elderly subjects to improve their systematic health have been reported [9, 14–16].

Although many evidences have been reported for the contribution of systemic exercise to improve immune function of elder people [4, 7], more moderate training or exercise is needed for them who cannot perform systemic exercise. Oral functional training, e.g. stimulating salivary grands and oral diadochokinesis [17], showed nutritional improvement [18] and also improvement of feeding function of elderly subjects [19]. Swallowing exercise showed improvement of salivary parameters, such as saliva flow rate, salivary pH at rest, and buffering capacity, suggesting the improvement of oral functions [20]. Thus, oral training is expected as an alternative exercise, which potentially contribute to immune functions of these elder people.

Indicators to objectively evaluate the effect of this training should be explored. Salivary secretory Immunoglobulin A (IgA) plays a central role in immune function in the oral cavity because it is a major effector of mucosal immunity by preventing submucosal invasion of pathogens [21]. Decreased secretory IgA secretion with aging is considered to be an indicator of attenuated immune system function, and leads to an increased incidence of respiratory tract infection [22]. There have been reports on recovery programs for attenuated immune systems comprising treatments such as oral functional exercise [23] and a combination of physical training and intake of lactobacilli [24]; however, data for highly older people (≥80 years) are not available.

The aim of this study was to investigate the effect of oral functional exercise on the immune function in older people who were over the age of 80 years. Stimulated saliva flow rate and salivary IgA were used as indicators as immune functions. Oral functional exercise were administrated for totally 6 weeks and the change of these parameters were observed.

## Methods

### Participants

This study was retrospectively registered as a University hospital Medical Information Network Clinical Trials Registry (UMIN-CTR) Clinical Trial (Unique trial Number: UMIN000028394) on 27/July 2017. This study was approved by the ethics committee at Ryotokuji University (No. 2506) and Oasis Geriatric Health Services facilities. The research was performed, following to the Declaration of Helsinki in 1995 (as revised in Brazil 2013). Informed consent was obtained from the volunteer participants and their families. All participants were recruited consecutively from Oasis Geriatric Health Services facilities. Subjects without any acute diseases, showing distinct consciousness and communication ability, were enrolled. The study was conducted from July to September 2013. Information on participant characteristics, dietary habits, diseases and medications was collected. Participants were recruited consecutively and subjects with xerostomia were excluded in this study. Written informed consent was obtained from each subject before participating in the study.

### Study protocol

Participants were enrolled in oral functional exercises before every meal (3 times per day) under the supervision of nurses. Participants were required to conduct self-massage; their tongues were used to press their orbicularis oris muscle and buccinators. These training were conducted within 15 min. For facilitating saliva secretion, participants were instructed to perform bilateral massage of three major glands, as described below (slightly modified from refs. [23, 25]):

- Parotid glands: four fingers from the forefinger to the little finger are placed on the cheeks and the upper molar area is moved back and forth (10 times).
- Sublingual glands: push up slowly the bottom area of chin using the thumbs of both hands (10 to 20 times).
- Submandibular glands: the inside soft part of the jaw is pressed with the thumbs, from below the ear to the tip of the chin, and sequentially pressed in four to five areas (5 times).

Subsequently, tongue and mouth exercises were conducted to simulate the orbicularis oris muscle and the mimetic muscles [23], as follows:

- the tongue is held out as far as possible and moved horizontally left and right (10 times).
- the tongue is held out as far as possible and moved vertically up and down to lick the nose and chin (10 times).
- plosive sounds (“Pa”, “Ta”, “Ka” “Ra”) were intensively pronounced in a loud voice up to 10 times.

The first oral exercise was conducted at 7:30–7:15 a.m. and breakfast was served at 7:30–8:00 a.m. Saliva samples were collected at 9:30–10:00 a.m., to eliminate diurnal variation, just after the mouth was rinsed out twice with natural water, and also to eliminate the acute stress caused by the exercise. The second oral exercise was conducted at 11:30–11:45 a.m. and lunch was served at 12:00–12:30 p.m. The third oral exercise was conducted at 11:30–11:45 a.m. and an evening meal was served at 12:00–12:30 p.m.

As baseline data, saliva samples were collected three times on different days when the oral functional exercise was not started, for the assessment of the daily variance of salivary parameters. The oral functional exercise were conducted totally 6 weeks. After 3 weeks of the oral functional exercise, saliva samples were collected. Subsequent 3 weeks of the oral functional exercise, saliva samples were collected again. Unstimulated whole saliva were collected using a sterilized cotton swab (Salikids; Saersted, Vümbrecht, Germany) for 2 min. We selected this product because of a string was attached to prevent from swallowing of the swab. The cotton was centrifuged at 626×g and 4°C for 15 min and the concentration of IgA was quantified using an enzyme-linked immunosorbent assay (ELISA). Stimulated saliva secretion flow rate (ml/min) was calculated based on the quantified saliva volume divided by 2 min, and the IgA secretion rate (ml/min) was calculated with the salivary secretion flow rate and IgA concentration.

### Measurement of salivary IgA

IgA concentration in the saliva samples was quantified using a human IgA ELISA Quantitation Kit (Bethyl Laboratories, Montgomery, TX). Ninety-six-well microtitre plates were coated for 1 h at room temperature (20–25 °C) with goat anti-human IgA (1:100 dilution) diluted with the coating buffer, 0.05 M carbonate-bicarbonate, at pH 9.6. The residual fluid was removed and washed five times with wash solution (50 mM Tris, 0.14 M NaCl, 0.05% Tween 20, pH 8.0). Blocking solution (50 mM Tris, 0.14 M NaCl, 1% bovine serum albumin pH 8.0) was added to the wells as a blocking agent. Then the plate was incubated for 30 min at room temperature and washed with wash solution as described. Saliva samples and IgA standards (Bethyl Laboratories) were added to each well. The plate was incubated for 1 h at room temperature and washed five times with wash solution. Horseradish peroxidase-conjugated goat anti-human IgA detection antibody (1:75,000 dilution) was added to each well. The plate was incubated again for 1 h at room temperature. The enzyme substrate TMB was added to each well after washing. The plate was developed in the dark at room temperature for 15 min. The reaction was stopped with 0.18 M H_2_SO_4_ stop solution. Absorbance was measured at a wavelength of 450 nm in an automated microplate reader (BioRad, Hercules, CA). Absolute concentrations (μg/ml) were calculated using a standard curve. The IgA secretion rate (μg/min) was calculated by multiplying the absolute concentration of IgA by stimulated saliva flow rate (ml/min).

### Data analysis

The averaged value of three saliva samples was used as the baseline data for each individual. Standard deviation (SD) and relative standard deviation (RSD), i.e. SD divided by the average, was also calculated. Salivary volume and IgA concentration with these flow rates before and after oral functional exercise were compared using the Wilcoxon matched-pairs signed rank test (two-tailed). Hierarchical clustering using Euclidian distance was used to extract individual-specific patterns of these data. The differences in each feature of prominent clusters were accessed and corrected by false discovery rate (FDR), accommodating multiple independent tests. The Wilcoxon rank-sum test (two-tailed) and the χ2 test (two-sided) were used for statistical analyses. *P* < 0.05 was considered statistically significant. JMP (ver. 12.0.1, SAS Institute, Cary, NC), MeV TM4 [26], R (ver 3.2.3, R-Foundation for Statistical Computation, Vienna, Austria) and GraphPad Prism (ver 5.04, GraphPad Software Inc., San Diego, CA) were also used.

## Results

### Salivary parameters

**Fig. 1:**
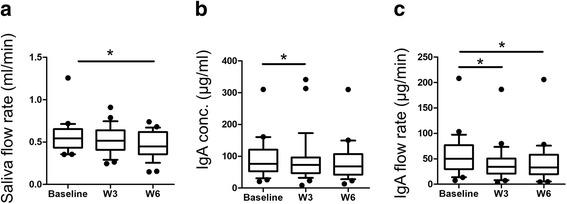
Box-whisker plots of stimulated salivary secretion rate, concentration of IgA and its secretion rate. The horizontal bars indicate 10, 25, 50, 75 and 90% of the data, and data <10% and >90% are depicted in the plots. The data at baseline and at 3 and 6 weeks after oral functional exercise are labelled Baseline, W3 and W6. Saliva flow rate (**a**), IgA concentration (**b**), and IgA flow rate (**c**). Wilcoxon matched-pairs signed rank test (two-tailed) was used. *P < 0.05

Overall, 30 participants whose age was 84.2 ± 8.5 years (mean ± SD) were enrolled into this study. Linearity of IgA quantification was *R* = 0.99 ± 0.029 (Pearson correlation, *n* = 3) using eight different concentrations between 0 μg/ml to 0.5 μg/ml. RSD of the salivary secretion volume and IgA baseline concentration was 16.1% and 35.6%, respectively. Data showing large differences (larger than 2-fold SD from the average) were treated as outliers and eliminated from the comparison between the data collected before and after exercise. Salivary secretion volume (*n* = 25) and IgA concentration (*n* = 27) data were used for statistical analyses. For the salivary secretion flow, data from participants who showed outlier data for either salivary secretion volume or IgA concentration were eliminated. Therefore, participants (*n* = 23) whose age was 83.4 ± 7.5 years were used for the analysis of salivary secretion flow. Stimulated saliva flow rate, IgA concentration and IgA secretion rate are summarized in Table 1. Wilcoxon matched-pairs signed rank test was used here. There was no significant difference between the stimulated show saliva flow rate at baseline compared to after 3 weeks of exercise (*Z*-value = −1.06, *P* = 0.29). In contrast, stimulated saliva flow rate after 6 weeks showed a significant decrease (*Z*-value = −2.14, *P* = 0.034). IgA concentration was significantly decreased after 3 weeks (*Z*-value = −2.12, *P* = 0.0035), while there was no significant difference after 6 weeks (*Z*-value = −1.39, *P* = 0.17). IgA flow rate was significantly decreased after 3 weeks (*Z*-value = −2.40, *P* = 0.017) and 6 weeks (*Z*-value = −2.13, *P* = 0.035; Fig. 1).

**Table 1:** Salivary parameters

Parameter	n	Baseline		After 3 weeks					After 6 weeks				
		Average	SD	Average	SD	*Z*-Value	*P*-value		Average	SD	*Z*-Value	*P*-value	
Stimulate saliva flow rate (ml/min)	25	0.563	0.185	0.515	0.168	−1.06	0.29		0.472	0.160	−2.14	0.034	*
IgA concentration (μg/ml)	27	91.23	58.42	87.49	75.66	−2.12	0.035	*	84.2	57.83	−1.39	0.17	
IgA secretion rate (μg/min)	23	54.94	42.87	42.42	37.12	−2.40	0.017	*	44.05	41.5	−2.13	0.035	*

IgA and SD indicated immunoglobulin A and standard deviation, respectively

*Z*-score and *P*-values were calculated by Wilcoxon matched-pairs signed rank test (both tailed)

### Clustering analysis

**Fig. 2:**
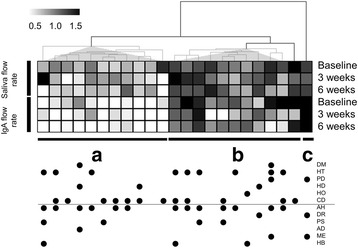
The clustering results of participants based on the pattern of stimulated saliva flow rate and IgA secretion rate. The quantified values were divided by the averaged value of each sampling point and colored in black and white for relative higher and lower values, respectively, compared with their average. The color bar (at the upper left) indicates the fold change of each quantified values (0.5, 1.0 and 1.5 indicated fold change). Data at a line indicates the values collected at a time point and data at a column indicates the values for each subject. The top three lines and the bottom three lines indicate the rate of salivary secretion and IgA secretion rate data, respectively. Dendrogram (presented in tree form) indicates the similarity of each column, yielded by clustering analysis. Elucidation distance was used for the clustering algorithm, e.g. columns showing similar data patterns were aligned closely. Diseases and the use of medication are represented by solid dots. Abbreviations are presented in Table 2. Prominent clusters are labelled **a**, **b** and **c**

Clustering analysis was conducted to understand the individual variation in the patterns of saliva and IgA flow of each participant, and it revealed three prominent clusters, indicating three representative patterns (Fig. 2). Participants with a lower baseline salivary secretion volume also had a lower baseline IgA concentration, and these values were further reduced after exercise (cluster A in Fig. 2). The clusters (clusters B and C) showed both a higher salivary secretion volume and IgA concentration at baseline. Based on these observations, we divided the individuals into two groups (cluster A vs clusters B + C), and evaluated the change in salivary parameters of each group. The Wilcoxon rank-sum test is used here. Cluster B + C showed a significant decrease in IgA concentration after 3 weeks (Wilcoxon matched-pairs signed rank test, *Z* = −2.12, *P* = 0.034) and in IgA secretion after 6 weeks (Wilcoxon matched-pairs signed rank test, Z = −1.88, *P* = 0.021). The other comparisons showed no significant change. These data also indicated that the stimulated salivary flow rate and IgA secretion were positively correlated.

When individual characteristics were compared between clusters A and B + C, only body mass index (BMI) showed a significant difference (*P* = 0.039; Table 2). However, no significant difference was observed using adjusted *P*-values. The participant in cluster A, who showed a lower salivary secretion rate and IgA concentration at baseline, had a higher BMI value of 23.4 ± 2.8 kg/m^2^, while those in clusters B + C showed a lower BMI value (20.5 ± 3.4 kg/m^2^). The effect of disease and the use of medication on the salivary parameters were evaluated (Table 3). Stimulated saliva flow rate was affected by Parkinson’s disease (The Wilcoxon rank-sum test, *Z* = 2.02, *P* = 0.04) and methyldopa (The Wilcoxon rank-sum test, *Z* = 2.02, *P* = 0.04), and the IgA concentration was affected antihypertensive agents (The Wilcoxon rank-sum test, *Z* = 2.03, *P* = 0.04). However, these significances disappeared using adjusted *P*-values calculated by FDR which controlled α error, by considering multiple independent univariate tests.

**Table 2:** Comparison of participants’ characteristics based on clustering analysis

Characteristics		Lower cluster (A)	Higher cluster (B + C)	Statistic Value	*P*-Value		Ajusted *P*-Value	
		(*n* = 11)	(*n* = 12)					
Age (years)		81.9 ± 8.1	84.7 ± 7.1	*Z* = 0.864	0.39		0.71	
BMI (kg/m^2^)		23.4 ± 2.8	20.5 ± 3.4	*Z* = 2.06	0.039	*	0.24	
Sex	Male	4	3	χ2 (1, n = 23) = 0.35	0.55		0.84	
	Female	7	9					
Drink milk	habit	3	3	χ2 (1, n = 23) = 0.015	0.90		0.94	
	no habit	8	9					
Eat yogurt	habit	3	3	χ2 (1, n = 23) = 0.015	0.90		0.94	
	no habit	8	9					
Exercise	habit	1	1	χ2 (1, *n* = 22) = 0.018	0.89		0.94	
	no habit	9	11					
False teeth	with	10	11	χ2 (1, n = 23) = 0.041	0.95		0.95	
	without	1	1					
Number of colds per year	0	1	2	χ2 (2, *n* = 16) = 1.04	0.59		0.84	
	1	3	4					
	2	1	5					
Number of times brush teeth per day	2	1	3	χ2 (2, n = 23) = 2.18	0.34		0.71	
	3	10	8					
	4	0	1					
Diabetes	with	1	1	χ2 (1, n = 22) = 0.011	0.88		0.94	
(DM)	without	10	8					
Hypertension	with	3	5	χ2 (1, *n* = 20) = 0.82	0.06		0.29	
(HT)	without	8	4					
Parkinson’s disease	with	0	1	χ2 (1, n = 20) = 0.82	0.26		0.62	
(PD)	without	11	8					
Heart diseases	with	2	1	χ2 (1, n = 20) = 0.099	0.66		0.88	
(HD)	without	9	8					
Hyperthyroidism	with	0	1	χ2 (1, n = 20) = 0.83	0.26		0.62	
(HO)	without	11	8					
Cerebrovascular disease	with	7	3	χ2 (1, n = 20) = 0.92	0.18		0.62	
(CD)	without	4	6					
Anti-hypertensive agent	use	6	3	χ2 (1, n = 20) = 0.15	0.58		0.84	
(AH)	no use	5	6					
Diuretics	use	0	1	χ2 (1, n = 20) = 0.83	0.26		0.62	
(DR)	no use	11	8					
Psychotropic drug	use	2	2	χ2 (1, n = 20) = 0.025	0.82		0.94	
(PS)	no use	9	7					
Antidepressants	use	1	0	χ2 (1, n = 20) = 0.69	0.35		0.71	
(AD)	no use	10	9					
Methyldopa	use	0	1	χ2 (1, n = 20) = 0.83	0.26		0.62	
(ME)	no use	11	8					
H2 blocker	use	1	2	χ2 (1, n = 20) = 0.33	0.41		0.71	
(HB)	no use	10	7					
Stimulated saliva flow rate (ml/min)		0.46 ± 0.11	0.59 ± 0.085	*Z* = 2.81	0.0049	**	0.039	*
IgA concentration (μg/ml)		57.7 ± 30.1	107 ± 27.8	*Z* = 2.89	0.0039	**	0.039	*
IgA secretion rate (μg/min)		26.4 ± 13.5	63.8 ± 16.2	*Z* = 3.42	0.00060	***	0.014	*

No participants had depression or epilepsy, and none used anti-hyperglycemic agents, antihistamine or α1-blocking drugs

Statistic value of χ2 test (two-sided) included degree of freedom, sample, size and *P*-values

*Z*-score and *P*-values were calculated by Wilcoxon rank-sum test (both tailed)

**Table 3:** Comparison of participant characteristics based on diseases present and medication use

Characteristics	Salivary flow (ml/min)				*Z*-Score	*P*-Value		Ajusted *P*-Value		IgA concentration (μg/ml)				*Z*-Score	*P*-Value		Ajusted *P*-Value		IgA secretion rate (μg/min)				*Z*-Score	*P*-Value		Ajusted *P*-Value	
	without/no use		with/use							without/no use		with/use							without/no use		with/use						
	Average ± S.D.	n	Average ± S.D.	n						Average ± S.D.	n	Average ± S.D.	n						Average ± S.D.	n	Average ± S.D.	n					
Diabetes	0.54 ± 0.11	21	0.56 ± 0.23	2	0.273	0.74		0.81		87.0 ± 44.6	21	122 ± 14.7	2	1.36	0.16		0.52		50.8 ± 30.9	21	66.8 ± 19.6	2	0.927	0.33		0.52	
Hypertension	0.51 ± 0.13	14	0.58 ± 0.084	9	1.23	0.21		0.50		81.8 ± 42.7	14	103 ± 45.0	9	1.10	0.26		0.52		46.0 ± 29.7	14	62.0 ± 29.7	9	1.291	0.19		0.52	
Parkinson’s disease	0.52 ± 0.11	21	0.69 ± 0.03	2	2.02	0.04	*	0.24		89.1 ± 44.6	22	112	1	0.678	0.45		0.54		50.9 ± 30.1	22	80.8	1	0.98	0.29		0.54	
Heart diseases	0.55 ± 0.11	19	0.49 ± 0.03	4	0.771	0.42		0.55		87.5 ± 37.5	19	102 ± 73.8	4	0.284	0.11		0.52		51.7 ± 26.8	19	54.9 ± 48.2	4	0.041	0.94		0.52	
Hyperthyroidism	0.54 ± 0.12	22	0.45	1	0.678	0.45		0.55		89.2 ± 44.7	22	111	1	0.528	0.55		0.60		52.3 ± 30.8	22	50.4	1	0	1.00		0.60	
Cerebrovascular disease	0.56 ± 0.11	12	0.51 ± 0.13	11	0.954	0.32		0.55		101 ± 42.9	11	80.2 ± 44.2	12	1.26	0.20		0.52		56.7 ± 28.8	11	48.1 ± 31.9	12	0.769	0.42		0.52	
Antihypertensive agent	0.54 ± 0.14	8	0.54 ± 0.11	15	0.161	0.85		0.85		63.1 ± 27.5	8	105 ± 44.8	15	2.03	0.04	*	0.48		40.0 ± 24.7	8	58.7 ± 31.4	15	1.323	0.18		0.48	
Diuretics	0.53 ± 0.13	20	0.63 ± 0.053	3	1.90	0.06		0.24		86.5 ± 39.9	21	127 ± 86.2	2	0.709	0.45		0.54		49.6 ± 27.2	21	79.6 ± 59.6	2	0.818	0.38		0.54	
Psychotropic drugs	0.55 ± 0.12	19	0.50 ± 0.12	4	0.689	0.46		0.55		93.6 ± 44.0	19	73.7 ± 45.5	4	0.771	0.42		0.54		54.6 ± 30.0	19	40.7 ± 31.9	4	0.771	0.42		0.54	
Antidepressants	0.55 ± 0.11	22	0.4	1	0.957	0.34		0.55		88.2 ± 43.9	22	133	1	0.258	0.23		0.52		52.2 ± 30.8	22	53	1	0.226	0.76		0.52	
Methyldopa	0.52 ± 0.11	21	0.69 ± 0.034	2	2.02	0.04	*	0.24		89.1 ± 44.6	22	112	1	0.678	0.45		0.54		50.9 ± 30.1	22	80.8	1	0.98	0.29		0.54	
H2 blocker	0.54 ± 0.12	20	0.52 ± 0.12	3	0.320	0.13		0.39		91.6 ± 45.0	20	80.3 ± 42.2	3	0.320	0.72		0.72		53.6 ± 31.0	20	43.1 ± 25.9	3	0.411	0.65		0.72	

Note: Participants showing high value ≥ average + 3 × SD were eliminated as outliers (one participant was eliminated for each comparison)

*Z*-score and *P*-values were calculated by Wilcoxon rank-sum test (both tailed)

## Discussion

### Comparison between our observation and other reports

We hypothesized that oral exercise for 6 weeks would contribute to improving immune system function, which induces increased saliva and IgA secretion. However, we did not observe these changes. This observation was inconsistent with previous studies that reported an increase in salivary IgA after moderate exercise in older people. For example, older people (ages 60 to 80 years) who participated in resistance training for 6 months showed a significant increase in salivary IgA, regardless their age or sex [27]. The daily activity levels of the older people in their 70s were classified using the number of steps, and participants with a higher number of steps secreted more saliva and IgA than those with fewer steps [5]. Resistance training and endurance training for 12 months in the older people (age 64.9 ± 8.4 years) increased salivary IgA [28]. An oral functional exercise program showed that participants who had 20 or more remaining teeth showed an improvement in oral function after 6 months of exercise [23].

There are three main differences between our study and other studies: (1) most of the participants in this study were older than the participants in other studies. All participants in our study were cared for in geriatric health services facilities and their daily activity level was expected to be lower than the participants who did not live in these facilities; (2) the duration of exercise was shorter than for other studies; and (3) whole body exercise was not used in our study, but rather the participants only performed oral functional exercises.

IgA concentration at younger ages (up to the 60s) increases, while it decreases in participants in their 60s; the IgA level in participants in their 60s was 92.9 ± 6.42 μg/ml [24]. The baseline value in this study was 91.23 ± 58.4 μg/ml, which is consistent with other reports showing IgA concentration is lower in older people than in those in their 60s.

**Fig. 3:**
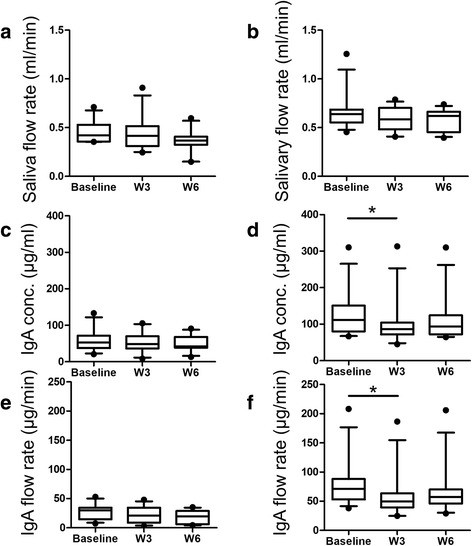
The change in salivary parameters for each group showing a lower stimulated saliva secretion rate and IgA secretion rate (cluster A in Fig. 2) and higher values (clusters B + C in Fig. 2). Salivary secretion flow rate (**a**) and (**b**), concentration of IgA (**c**) and (**d**), and the secretion rate of IgA (**e**) and (**f**). Panels **a**, **c** and **e** present the data in cluster A (*n* = 11) and panels **b**, **d** and **f** present the data in the clusters B + C (*n* = 12). Only the data without any missing value was used. Wilcoxon matched-pairs signed rank test (two-tailed) was used. *P < 0.05

Oral functional exercise for 3 months in the older people (age, 77.9 ± 6.5 years) showed a drastic increase in oral lip closure and the amount of oral diadochokinesis. In addition, a repetitive saliva swallowing test also showed an increase in the amount of swallowing for the participants who had a lower amount of swallowing (≤3), while the participants who showed more swallowing (≥4) had no significant increase. It was concluded that the 3-month duration of the training was short and not enough to contribute to the recovery of their immune system [19]. A shorter duration of oral functional exercise in our study probably might not contribute to an improvement of the immune system. In addition, all salivary parameters were reduced after the training programs (Fig. 3), which was unexpected. Because both the immune system and acute stress contribute to increased IgA [20], a reduction in acute stress is one possible explanation while saliva samples were collected 1.5 h and after breakfast.

The observed salivary parameters, especially IgA concentration, showed large individual variances at baseline. One possible reason was the use of cotton for saliva collection, which may have caused the differences in salivary secretion; although small variations in IgA concentration resulting from differences in chewing forces have been reported [29]. Stress can also influence IgA concentrations [30]. Although salivary IgA is widely used to evaluate immune function, the profiling of other substance, such as, lactoferrine showing antimicrobial activity, would help identify subjects’ specific patterns [31]. The use of simultaneous molecule profiling technique [32, 33] is also would contribute to understand the relationship between salivary parameters and factors that influence the parameters.

### Clustering analysis

Clustering analyses were conducted to explore new subtype of the subjects based on the similarity of multiple observations. The analyses resulted in BMI was a primary parameters characterizing salivary parameters including both salivary flow rate and IgA concentrations (Fig. 2 and Table 2). Each cluster included subjects shows BMI value of 23.4 ± 2.8 and 20.5 ± 3.4 kg/m^2^. Both of them were not in overweight range. Among old women, overweight BMI was associated with slightly reduced mortality among those in poor health and underweight BMI was significantly associated with increased mortality [25]. Physical function was observed for the old person showing decline of BMI along with age [22]. These relationships between BMI and health conditions are considered as possible reasons for the association with BMI and immune functions, however, our observation should be confirmed by larger cohort to more confident conclusion.

## Conclusions

There were some limitations to our study. Our participants showed large individual variances in salivary parameters; thus, enrolling a larger number of participants would be necessary to make stronger conclusions. More variables including salivary markers should be collected to identify the factors causing individual differences in salivary parameters. A clustering technique revealed that all participants were grouped into two groups based on the patterns of these salivary parameters. A significant change after oral functional exercise was observed only in the group showing a higher salivary secretion rate and IgA concentration at baseline. This is the first report to reveal such individually specific salivary parameter patterns in older people over the age of 80 years.

## Abbreviations

BMI: body mass index
ELISA: enzyme-linked immunosorbent assay
FDR: false discovery rate
IgA: Immunoglobulin A
RSD: relative standard deviation
SD: standard deviation
URTI: upper–respiratory tract infections
